# Promoting sustainable physical activity among middle-aged Iranian women: a conceptual model-based interventional study

**DOI:** 10.1186/s12905-020-01152-w

**Published:** 2021-01-02

**Authors:** Mohammad Shariati, Houra Pourrajabali Astaneh, Leila Khedmat, Farnaz Khatami

**Affiliations:** 1 Community Medicine Department, Tehran University of Medical Sciences, Tehran, Iran; 2 Family Medicine Department, Ziaeian Hospital, Tehran University of Medical Sciences, Tehran, Iran; 3 Health Management Research Center, Baqiyatallah University of Medical Sciences, Tehran, Iran

**Keywords:** Physical activity, Middle-aged women, Motivational model, Behavioral modification

## Abstract

**Background:**

Physical inactivity enhances the risk of adverse health conditions such as non-communicable diseases, morbidity, and mortality among middle- and older-aged population. This study is aimed to design, implement, and evaluate a conceptual model of physical activity (PA) promotion among middle-aged Iranian women (MAIW).

**Methods:**

An interventional study was conducted with 80 women aged between 30 and 59 years in intervention and control groups during 2018–2019. The subjects referred to health centers were selected by the available convenience sampling method. Data collection tools to assess the MAIW' PA level (metabolic equivalent tasks (MET)-min/week) included face-to-face interviews, body mass index (BMI) measurements, the four-question form of PA vital signs in the framework of Iran's Package of Essential Non-communicable (IraPEN) program, and the questionnaire is based on the Health Belief Model (HBM) constructs.

**Results:**

The intervention led to a three-fold increase in the average PA (from 280.63 to 927.70 MET-min/week) of the intervention group. Although no significant difference in the BMI between both groups was found before the intervention, this educational program decreased the mean BMI from 30.36 to 28.83 kg/m^2^ (*p* = 0.01). After the intervention, the values of HBM-based perceived sensitivity/severity and self-efficacy were increased from 62.09 to 71.03% and from 27.01 to 83.15%, respectively (*p* < 0.0001). There were no significant differences in the cue to action and perceived benefits and barriers after the intervention.

**Conclusion:**

The developed model by increasing the motivation of MAIW could remarkably improve the PA level with a decrease in their BMI.

*Trial register* Iranian Registry of Clinical Trials (IRCT): IRCT20200717048124N1 at 2020-08-05, retrospectively registered.

## Background

Nowadays, the leading causes of morbidity and mortality in the globe are the development of chronic non-communicable diseases (NCDs) such as cardiovascular diseases (CVDs), overweight, obesity, diabetes, hypertension, and cancer types [1]. Lifestyle modification programs using an integration of effective interventions including regular physical activity (PA), proper nutrition, and stress management can significantly decrease the long term risks associated with modern chronic diseases [1–4]. Physical inactivity is rapidly increasing in low- to middle-income countries [4]. There is a considerable variety of physical inactivity in different geographical regions of the world because this risk factor is highly influenced by age, gender, health status, self-efficacy (SE), and motivation [3, 5]. Based on the data disseminated by the World Health Organization, the inadequate PA caused 9% of the global deaths [6]. Furthermore, regular physical inactivity has been the main risk factor for 21–25% of breast and colon cancers, 27% of diabetes, and 30% of CVDs [7]. CVDs are the most critical consequence of physical inactivity [8]. In 2016, the US healthcare system spent over $315.4 billion on CVDs and stroke caused by a sedentary lifestyle [9]. According to the information released about the disadvantages of a sedentary lifestyle, most Iranians are in a state of extreme immobility. Findings show that more than 80% of Iran's population are physically inactive (less than 90 min per week for 3 months) [10]. Since the Iranian people are moving towards aging, it is not far-fetched to predict the increase in burden of chronic diseases and their risk factors [11]. It is anticipated that by 2050, approximately 20% of the worldwide population will be women older than 50 years old [12]. Besides, according to the Population and Housing Censuses 2016, 8.5 million 40-64-year-old women with very little PA are living in Iran [13]. The health status of middle-aged people in the society can be effectively improved using an integrated behavior change model as a visible and replicable interventional tool through implementing empirical and theoretical aims, program development, feedback, and monitoring [14]. These interventions may involve a large number of population groups according to the integration of demographic parameters such as gender, age, marital and parenting status, and socioeconomic situation. Hence, the in-depth understanding and knowledge of the unique characteristics of individuals (such as values, motivators, preferences, and challenges) are necessary to plan an efficient intervention for promoting the PA level [15]. Accordingly, designing behavior change programs and promoting PA allow middle-aged Iranian women (MGIW) to understand the impact of their behavior on health and to make decisions about having a healthy life. Consequently, the present study is aimed to choose and assess a suitable and indigenous model to promote the PA of MAIW.

## Methods

### Study design and subjects

An interventional quasi-experimental study for three months between December 2018 to April 2019 was performed to promote the PA level among MAIW residing in Tehran. Eighty Iranian women within 30–59 years old using the available convenience sampling method were chosen among people who referred to the Health Centers of Shahabadi and Abouzar (Tehran, Iran). One of these health centers was allocated to the intervention group and the other to the control group. This design was conducted due to prevent contamination bias, in order not to inform the control group about the intervention as there was no possibility of blinding. The sample size (*n*) was calculated using the following formula (Eq. 1):

1 $$n = \frac{{\left( {Z_{1} - \frac{\alpha }{2} + z1 - \beta } \right)^{2} \left( {\sigma_{1}^{2} + \sigma_{2}^{2} } \right)}}{{\left( {\mu_{2} - \mu_{1} } \right)^{2} }}$$

According to the significance level α = 0.05, (${Z}_{1-\alpha/2}$) = 1.96, β or type II error = 0.2, power 1-β = 0.90, (${Z}_{1-\beta }$) = 1.28, SD_1_ = 7.3, SD_2_ = 4.8, μ_1_ = 2.7, and μ_2_ = 7.5, the total sample size was calculated to be 70. Based on the attrition risk of 10%, the sample size was reached to 40 subjects in each group. Only the statistical consultant in this study was blinded.

### Inclusion and exclusion criteria

The inclusion criteria for all MAIW were as follows: being in a middle-age group (30–59 years), having at least a middle-school literacy level, having an inactive lifestyle (less than 90 min of moderate-intensity PA (e.g., brisk walking, and jogging) per week according to the instructions of the PA promotion program of Iran Package of Essential Non-Communicable Disease (IraPEN), and completing the consent form to participate in the study. In 2014, IraPEN was developed by the Iranian Ministry of Health and Medical Education to provide universal health coverage for the prevention and care of NCDs and the delivery of mental health services. This program as part of the national health transformation instruction was mainly developed to control the emerging epidemic of NCDs. The exclusion criteria were limited to having some chronic diseases such as CVDs, uncontrolled diabetes and hypertension, as well as chronic kidney disease.

### Behavioral change model assessment

Several behavioral change models, including trans-theoretical model (TTM), precede-proceed model (PPM), health belief model (HBM), beliefs, attitudes, subjective norms and enabling factors (BASNEF) model, and health promotion model (HPM), were considered to evaluate the best PA-promoting model after the literature review and unstructured face-to-face interview with the panel of experts (PEs). The PEs included twenty specialists (in the fields of sports medicine, community medicine, psychiatrists, health education, and genetics) with long-term executive backgrounds related to the health system management and program directors. The study's purpose and content and how to record and collect information were first explained before the interview. Then, the experts with open questions were asked to comment on the PA behavior change models, the most important factors hindering PA in MAIW, and possible ways to improve their PA level [see Additional file 1]. The interviewees were completely free to express their views within about 45 min. All the recorded and written data were consequently summarized without any individual judgment. The validity of this questionnaire was confirmed by internal experts. Results of the experts' comments showed that the HBM was the best initial model to improve PA in MAIW. Accordingly, individuals in the intervention group completed the HBM-based questionnaire to find the most important solutions in promoting MAIW's PA. The HBM-based questionnaire has been validated in Persian [16]. Demographic indicators and physical activity levels of the control group were also assessed at the begging and end of the study. This group just received some PA-related brochures and routine recommendations available in healthcare centers.

### Intervention program: design and implementation

**Fig. 1:**
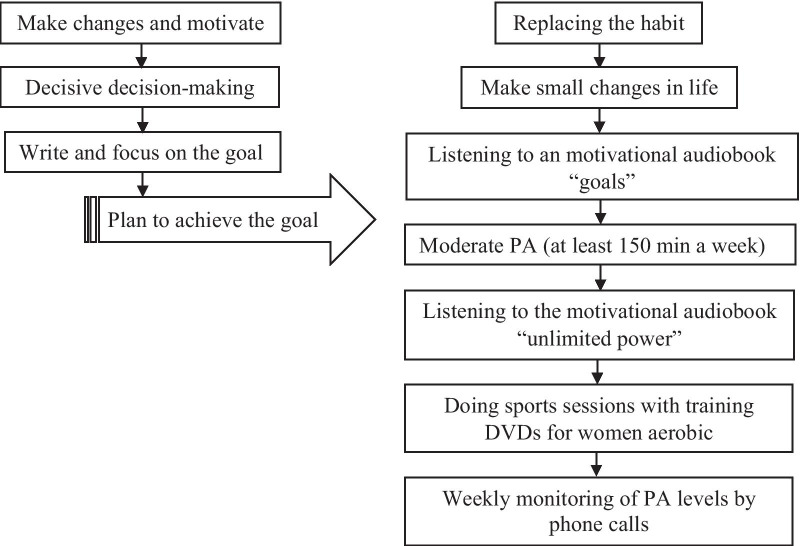
A developed pre-instruction to promote the PA level in MAIW based on the integration of IraPEN and HBM

The intervention was both face-to-face and virtual meetings. Once a month, a face-to-face intervention session was fulfilled for an hour and a half by mentioning questions and answers. For the intervention group, an HBM-based questionnaire in the first face-to-face session was completed. All members in the intervention group were supported by presenting 80-min tutorial videos concerning useful knowledge of aerobic and resistance exercises. In this DVD, exercise movements using stretch band, cardio fit ball, and 1–2 kg dumbbells were trained to strengthen muscles and keep the body in shape from beginner to advanced level. A social group called “Success” was made by registering all individuals in the intervention group and followed by the virtual intervention for three months. The virtual intervention was also held three times a week for 180 min. A total of 36 virtual sessions were held for 108 h. In the first and second months of the intervention, the voice book of "goals! how to get everything you want faster than you ever thought possible" written by Brian Tracy, and the voice file of “unlimited power: the new science of personal achievement” from Anthony Robbins were presented, respectively. In the second and third face-to-face sessions, an algorithm was designed after highlighting key points of these books based on the following steps: (i) inspire change and motivate excellence, (ii) decisive decision-making, (iii) write goals clearly, (iv) focus on the goal, and (v) plan to achieve the goal. Finally, the practical program to achieve the goal was implemented based on comprehensive studies of the health system and available resources (Fig. 1). At the end of the third month of the intervention, the PA and body mass index (BMI) levels of MAIW were assessed in the intervention and control groups. Throughout the study, in addition to the intervention sessions, participants by telephone were weekly contacted by telephone to examine how they understood the intervention and to answer their questions.

### Data collection

#### Demographic and anthropometric measures

The demographic data (e.g., age, marital status, literacy, etc.) of MAIW in both groups were collected through a face-to-face interview. The participates' height was determined without shoes in a standing position by a stadiometer to the nearest 0.5 cm, whereas the shoulders were in a normal state. The body weight of MAIW with wearing light clothes and no shoes or socks was measured and recorded using a SECA 768,611 balance scale (Seca™, Seca Vogel & Halke GmbH & Co., Hamburg, Germany) with an accuracy of 0.1 kg. The BMI was calculated by dividing the weight (in Kg) by the square of measured height (in m).

#### IraPEN-based assessment of physical activity

All the participants completed a four-question form of PA vital sign according to the IraPEN instruction [see Additional file 2]. In this checklist, the median (slow running or brisk walking) and intense (brisk running or brisk walking) PA levels are asked within a week. This parameter is expressed in metabolic equivalent tasks (MET)-min/week. One MET is usually considered to be 3.5 mL/min/kg resting oxygen consumption. The weekly total PA is obtained from the daily sum of the average (600–1500 MET-min/week) and vigorous (> 1500 MET-min/week) PA amounts during a week. According to the IraPEN guidelines, adults with a standard PA do at least 150 min of moderate aerobic PA or do at least 75 min of vigorous aerobic PA throughout the week. A person is considered sedentary if her/his PA with moderate intensity is less than 90 min. The validity of the questionnaire was confirmed by fifteen experts.

#### Health Belief Model (HBM) questionnaire-based data

MAIW assigned to the intervention group filled up the validated HBM 96-item questionnaire in Persian before and after the designed intervention [16]. This questionnaire is based on six constituent constructs including perceived sensitivity (PSS) and severity (PSV) (17 items), cue to action (CA, 15 items), perceived benefits (PBF) and barriers (PBR) (54 items), and SE (10 items). A four-point Likert scale was used for most items (PSS, PSV, PBR, and CA) within a score range from 1 (strongly disagree) to 4 (strongly agree). Also, a five-point Likert scale was employed for the SE sub-group, ranging from 1 “not confident at all” to 5 “very confident”. Based on the items available in PSS and PSV sub-groups, the minimum and maximum score values are 17 and 68, respectively, while the PBF and PBR have a maximum score of 216 and a minimum score of 54. According to the Likert scale ranges and the number of items in the questionnaire, the lowest and highest scores for CA and SE are 15 and 60, as well as 10 and 50, respectively [16]. The internal consistency reliability of the HBM questionnaire was assessed using Cronbach’s alpha coefficient (CAC). High reliability was recognized by estimating CACs of 0.72–0.85 for the different constructs in the questionnaire. The content validity was also determined using 10 experts in the field of community health and medicine. The questionnaire was filled out face to face in an hour. The collected data before the intervention were statistically analyzed to determine with which part of the HBM questionnaire did the intervened individuals have the most difficulty.

### Statistical analysis

Statistical analyses were performed using the Statistical Package for Social Sciences (version 22.0; SPSS Inc., Chicago, IL, USA). In the descriptive analysis, means and standard deviations were presented for normally distributed continuous data. The comparison of categorical and continuous variables between control and intervention groups were respectively performed by Fisher's exact and independent-samples *t*-tests. Statistical significance was set at *p* < 0.05.

## Results

Table 1 shows some demographic characteristics of MAIW in the control and intervention groups. There was no loss in any group in this study. The age of participants in the control and intervention groups was 41.5 and 41.4 years, respectively. Most individuals in both groups were married with an educational level of diploma and above (Table 1). There were no significant differences in age (*p* = 0.9), as well as marital and educational status (*p* = 0.3) between the two groups of control and intervention. Therefore, the two groups were homogeneous concerning the investigated demographic characteristics.

**Table 1:** Some demographic data of MAIW in the control and intervention groups

Demographic characteristics	Control group (n = 40)	Intervention group (n = 40)	*p* value
Age (years old)	41.50 ± 7.85	41.40 ± 7.54	0.90
Marital status [n (%)]			
Married	39 (97.5)	37 (92.5)	0.61
Single/Separated	1 (2.5)	3 (7.5)	
Education level [n (%)]			0.30
Under diploma	12 (30.0)	13 (32.5)	
Diploma and above	28 (70.0)	27 (67.5)	

The data collected from the HBM questionnaire before the intervention mentioned that the lowest and highest scores obtained by the intervened MAIW were related to the SE (27.01) and CA (81.30), respectively (Table 2). Therefore, the HBM in this MAIW group will be effective on their SE. Accordingly, the most important problems of the members (low SE and lack of motivation) in the design of the main model were taken into account. Table 2 reveals that the mean PSS-PSV and SE scores after the intervention has significantly increased from 62.09 to 71.03 and from 27.01 to 83.15, respectively (*p* < 0.0001). However, no significant increase in CA and PBF-PBR scores was found (Table 2).

**Table 2:** The HBM scores of MAIW in the intervention group before and after the PA promoting intervention

HBM constructs^†^	Total score	Intervention program^‡^		Significance level (*p* value)
		Before	After	
PSS-PSV	66.73 ± 11.00	62.09 ± 6.03^b^	71.03 ± 12.76^a^	< 0.0001
CA	81.91 ± 4.99	81.30 ± 4.49	82.48 ± 5.42	0.57
PBF-PBR	70.33 ± 1.00	68.92 ± 8.96	71.63 ± 1.09	0.30
SE	55.08 ± 3.10	27.01 ± 6.86^b^	83.15 ± 1.68^a^	< 0.0001

^†^HBM, Health belief model; PSS, perceived sensitivity; PSV, perceived severity; CA, cue to action; PBF, perceived benefits; PBR, perceived barriers; SE, self-efficacy

^‡^a, and b, are significant statistical letters

**Fig. 2:**
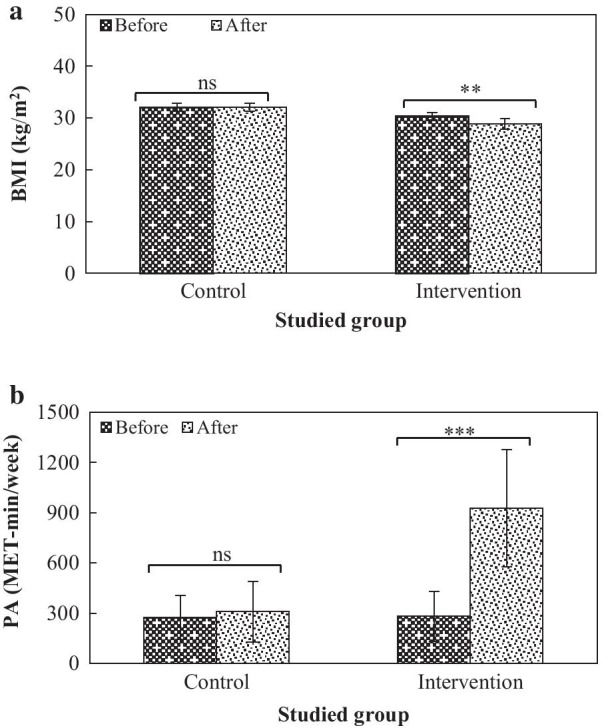
The BMI (**a**) and PA (**b**) levels of MAIW in control and intervention groups before and after the health promotion program (ns, non-significant, ** and *** are significant at *p* values of < 0.01 and < 0.001, respectively)

Before the intervention program, there was no significant difference in BMI values between control and intervention groups (*p* = 0.1). Although no significant difference in BMI of the control group was observed, the BMI value of the intervention group was significantly reduced from 30.36 to 28.83 kg/m^2^ (Fig. 2a). Therefore, the effect of the HBM program on the BMI (1.52 kg/m^2^) reduction in intervened women was significant compared to the control (*p* = 0.01). Moreover, no significant difference in the PA level between both groups was found before the intervention implementation (*p* = 0.8). Figure 2b illustrates that the PA level in the intervened group was remarkably increased from 280.63 to 927.70 MET-min/week (*p* < 0.001). However, the PA increase in the control group from 275.62 to 308.75 MET-min/week was non-significant. Thus, a more than threefold increase was recorded in this group after the intervention (647.07 MET-min/week), while the PA increase in the control group was only 33.13 MET-min/week. Consequently, the increased PA in the intervention group was significantly higher than the control group (*p* < 0.001).

## Discussion

Women at older ages usually have a lower PA level compared to men. PA-related behavioral changes in women typically occur at the time of menopause and beyond. The insufficient PA is considered as the main cause of many chronic diseases such as cardiovascular, respiratory, musculoskeletal, and metabolic diseases [4, 6, 11]. Thus, there is a necessity to design appropriate behavioral models to change the PA pattern in women of this age group. The HBM was chosen as an appropriate model to promote the PA level in MAIW after reviewing the literature about efficient models on behavioral changes. This model is relatively a short-term intervention in prevention programs. People in this practical model should initially feel threatened by the increased risk of BMI and chronic diseases (PSS) and subsequently perceive the risk severity and the significance of its various related-complications on physico-mental and socio-economic aspects (PSV). Individuals then would be able to observe optimistic signs received from their surroundings (CA) and to believe in the helpfulness and efficiency of prevention programs (PBF) with low obstacles to behavior change [17, 18]. The assessment of HBM components before and after the intervention showed that there was no significant difference in CA and PBF-PBR. However, the intervention implementation significantly changed the PSS-PSV and SE. These results were consistent with studies in which educational interventions led to increased awareness of the health effects of regular PA [19–21].

Results showed that the increased motivation and SE of MAIW could promote PA levels with a reduced BMI. Earlier, virtual training through a web- and mobile phone-based intervention could improve the PA level in Australian middle-aged males [22]. These researchers also emphasized that virtual training should be given priority over paper training due to high availability and increasing use of mobile phones and social networks [22]. In a meta-analysis study, the role of interventional training in increasing the PA level of more than 22,500 adults with chronic diseases was examined. Results showed the higher average PA in the intervention group compared to the control one (48 min of PA per week or 945 steps per day). In most studies, there was no correlation between PA levels and demographic data of age, sex, and socio-economic status. However, factors such as the design of regular programs, continuous monitoring, practical considerations of job conditions, and adequate available time played the most important role in the steady implementation of the PA-promoting program [23]. A high association between high levels of PA over 15 years and autonomous motivation and SE of ninety American MAW was reported. Also, 61% of these people tended to make friends with highly-active individuals [24]. The effect of aerobic training and pedometer-based counseling in a 90-day interventional study was assessed to increase the PA level by reducing anthropometric factors. The pedometer-based counseling effectively increased the daily number of steps among Brazilian MAW, while aerobic training contributed to reducing their body weight loss [25]. Also, the PA level of obese American MAW was significantly promoted due to their increased motivation for lifestyle changes using the interventionist-led group intervention for three months [26].

## Conclusion

Reviewing the literature and doing interviews with experts showed that the lack of motivation was one of the most significant factors involved in MAIW's inactivity. In this study, an educational intervention to increase the motivation level was developed based on the PA promotion model. The designed model resulted in a significant increase in the PA level and a decrease in BMI. Despite the difficulty of PA increase in MAIW, this healthy lifestyle approach in the short-term period was well achievable by increasing motivation and emphasizing on fitness, beauty, and attractiveness following ongoing PA in this population group. In general, a permanent behavior change cannot be made without constantly reinforcing it. Therefore, positive behavioral changes occurred in individuals should be encouraged and reinforced in the short term (once a week to once a month) to long term (once every 6 months to once a year) periods. Accordingly, the present study has some limitations. The selected design might affect internal validity as the study design was an interventional quasi-experiment without randomization. On the other hand, it has not been followed for longer than three months. Inactivity like chronic diseases is multifactorial and needs follow-up. Simultaneous implementation of social interventions such as increasing the number of women's parks, clubs, and available exercise environments would be appropriate to improve the PA level of MAIW. Since one of the limitations of the present study was air pollution in Tehran, a metropolitan city, the use of indoor exercise environments, with controlled and invariable conditions, is recommended to train in a bustling or quiet fitness space. Also, a motivational follow-up system may efficiently improve research findings with the surveillance of MAIW's PA through health care providers or online systems.

## Supplementary Information

**Additional file 1**. Experts’ open questions to comment on the PA behavior change models.

**Additional file 2**. Four-question of PA vital sign according to the IraPEN instruction.

## Data Availability

The datasets used and/or analyzed during the current study are available from the corresponding author on reasonable request.
