# Supplementary material for: Promoting sustainable physical activity among middle-aged Iranian women: a conceptual model-based interventional study
Source: BMC Womens Health. 2021 Jan 2;21:1. doi: 10.1186/s12905-020-01152-w (PMC7777291; doi:10.1186/s12905-020-01152-w)
Supplement: Supplementary file 1 — Additional file 1. Experts’ open questions to comment on the PA behavior change models. [file 12905_2020_1152_MOESM1_ESM.docx]

**Experts questionnaire**

1. What do you think about behavioral change models?
2. What are the factors that inhibit physical activity in middle-aged Iranian women?
3. What are the strategies to improve physical activity in middle-aged women?
