# Supplementary material for: Promoting sustainable physical activity among middle-aged Iranian women: a conceptual model-based interventional study
Source: BMC Womens Health. 2021 Jan 2;21:1. doi: 10.1186/s12905-020-01152-w (PMC7777291; doi:10.1186/s12905-020-01152-w)
Supplement: Supplementary file 2 — Additional file 2. Four-question of PA vital sign according to the IraPEN instruction. [file 12905_2020_1152_MOESM2_ESM.docx]

**Physical activity questionnaire**

1. How many minutes of moderate activity do you do per day? (such as jogging or brisk walking)
2. How many days a week do you do moderate activity? (such as jogging or brisk walking)
3. How many minutes a day of strenuous activity do you do? (such as run fast or walking too fast)
4. How many days a week are you active (such as run fast or walking too fast)?
